# Transcription Factor ChREBP Mediates High Glucose-Evoked Increase in HIF-1α Content in Epithelial Cells of Renal Proximal Tubules

**DOI:** 10.3390/ijms222413299

**Published:** 2021-12-10

**Authors:** Aleksandra Owczarek, Katarzyna B. Gieczewska, Robert Jarzyna, Zuzanna Frydzinska, Katarzyna Winiarska

**Affiliations:** 1 Department of Metabolic Regulation, Faculty of Biology, Institute of Biochemistry, University of Warsaw, 02-096 Warsaw, Poland; aowczarek92@biol.uw.edu.pl (A.O.); rjarzyna@uw.edu.pl (R.J.); z.frydzinska@student.uw.edu.pl (Z.F.); 2 Department of Plant Anatomy and Cytology, Faculty of Biology, Institute of Experimental Plant Biology and Biotechnology, University of Warsaw, 02-096 Warsaw, Poland; kat.gieczewska@biol.uw.edu.pl

**Keywords:** hypoxia-inducible factor-1 (HIF-1), high glucose, carbohydrate response element binding protein (ChREBP), NAD^+^/NADH ratio, sirtuin 1, renal proximal tubules

## Abstract

Hyperglycemia/diabetes appears to be accompanied by the state of hypoxia, which especially affects kidneys. The aim of the study was to elucidate the mechanism of high glucose action on HIF-1α expression in renal proximal tubule epithelial cells. The research hypotheses included: (1) the participation of transcription factor ChREBP; and (2) the involvement of the effects resulting from pseudohypoxia, i.e., lowered intracellular NAD+/NADH ratio. The experiments were performed on HK-2 cells and primary cells: D-RPTEC (Diseased Human Renal Proximal Tubule Epithelial Cells—Diabetes Type II) and RPTEC (Renal Proximal Tubule Epithelial Cells). Protein and mRNA contents were determined by Western blot and RT-qPCR, respectively. ChREBP binding to DNA was detected applying chromatin immunoprecipitation, followed by RT-qPCR. Gene knockdown was performed using siRNA. Sirtuin activity and NAD^+^/NADH ratio were measured with commercially available kits. It was found that high glucose in HK-2 cells incubated under normoxic conditions: (1) activated transcription of HIF-1 target genes, elevated HIF-1α and ChREBP content, and increased the efficacy of ChREBP binding to promoter region of *HIF1A* gene; and (2), although it lowered NAD^+^/NADH ratio, it affected neither sirtuin activity nor HIF-1α acetylation level. The stimulatory effect of high glucose on HIF-1α expression was not observed upon the knockdown of ChREBP encoding gene. Experiments on RPTEC and D-RPTEC cells demonstrated that HIF-1α content in diabetic proximal tubular cells was lower than that in normal ones but remained high glucose-sensitive, and the latter phenomenon was mediated by ChREBP. Thus, it is concluded that the mechanism of high glucose-evoked increase in HIF-1α content in renal proximal tubule endothelial cells involves activation of ChREBP, indirectly capable of *HIF1A* gene up-regulation.

## 1. Introduction

Hypoxia-inducible factors (HIFs), namely HIF-1, HIF-2, and HIF-3, are crucial for the adaptation to hypoxic conditions, and they regulate metabolic shift from the oxidative way of energy recruitment to anaerobic glycolysis. HIFs directly control the expression of genes encoding enzymes and other proteins essential for effective glucose utilization in the latter process, including glucose transporter GLUT1, pyruvate dehydrogenase kinase 1 (PDK-1; it inhibits pyruvate dehydrogenase-catalyzed conversion of pyruvate to acetyl-CoA), hexokinase, and phosphofructokinase 1 [1,2]. Moreover, HIF-1 was reported to accelerate the rate of hepatic [3,4] and, according to our latest findings [5], also renal gluconeogenesis, contributing to the increase in the whole body glucose supply.

On the other hand, which seems of special interest, hyperglycemia/diabetes appears to be accompanied by the state of hypoxia (cf. Reference [6] for review). In diabetic kidneys, hypoxia probably results mainly from elevated oxygen consumption, being a consequence of glomerular hyperfiltration and increased activity of sodium-dependent glucose transporters (SGLTs) and Na^+^/K^+^ ATPase [1,7]. Moreover, hyperglycemia itself might also cause a decline in the intracellular NAD^+^/NADH ratio, the state sometimes defined as “pseudohypoxia”, leading to many deleterious metabolic consequences (cf. References [8,9] for review), including its inhibitory effect on sirtuins, a family of seven enzymes with deacetylase activity [10].

HIF-1, the most important of HIFs in renal proximal tubules [2,11], is a heterodimer composed of subunits: HIF-1α and HIF-1β. The subunit β, responsible for the binding of the transcription factor to HRE (hypoxia response element) sequence of target genes, is expressed constitutively. In contrast, the level of the regulatory subunit α is strictly regulated and HIF-1α is guided to be degraded in proteasome when oxygen supply is sufficient [2,11]. In addition to regulating the stability of HIF-1α by oxygen concentration, the ultimate activity of HIF-1 is also controlled by a variety of other factors, including those affecting transcription, translation initiation, stability and nuclear translocation of HIF-1α subunit, and, finally, functional HIF-1 dimer activity (cf. Reference [12] for review). Sirtuins, especially sirtuin 1 (SIRT1), are also supposed to modulate HIF-1α stability via its deacetylation [13,14,15,16,17].

The regulation of HIF-1 activity under diabetic conditions is an especially intriguing issue. Generally, it is accepted that hyperglycemia tends to suppress the cellular response to hypoxia, including HIF-1 activity [6,9,18]. However, there are some exceptions, e.g., elevated expression of HIF-1α was found in mesangial cells of renal glomeruli exposed to high glucose or isolated from animal models of diabetes [19,20,21,22,23]. One of the most inspiring explanations of this phenomenon is the contribution of carbohydrate response element binding protein (ChREBP)—the transcription factor was found to bind to the ChoRE (carbohydrate response element) sequence present in the regulatory region of *HIF1A* gene [19,24].

ChREBP was initially described as a regulator of lipogenesis in liver and adipose tissue but is now considered to be a key whole-body glucose sensor, expressed in various organs, including kidneys [25,26]. ChREBP protein structure contains two important regulatory domains: a low glucose inhibitory domain (LID) and a glucose-response activation conserved element (GRACE). Activation of GRACE domain by glucose metabolites promotes ChREBP transcriptional activity and its binding to ChoREs localized inside ChREBP target genes, e.g., those encoding pyruvate kinase, fatty acid synthase, acetyl-CoA carboxylase, and stearoyl-CoA desaturase. Moreover, recently, the existence of the LID-lacking, i.e., highly active, isoform ChREBPβ was reported [25,26].

Surprisingly, the effect of hyperglycemia/diabetes on HIF-1α expression in epithelial cells of renal proximal tubules is still poorly understood. As a matter of fact, the data seem to be contradictory [27,28,29,30]. Thus, the aim of the present study was to elucidate the mechanism of high glucose action on HIF-1α expression in human renal proximal tubule epithelial cells. The hypotheses to be verified included: (1) the participation of the transcription factor ChREBP; and (2) the involvement of the effects resulting from the state of pseudohypoxia, i.e., the lowered intracellular NAD^+^/NADH ratio. Moreover, we decided to analyze diabetes-evoked differences in the cellular response to high glucose, determining HIF-1α expression in primary cells: D-RPTEC (Diseased Human Renal Proximal Tubule Epithelial Cells—Diabetes Type II) and RPTEC (Renal Proximal Tubule Epithelial Cells).

## 2. Results

### 2.1. High Glucose Results in Elevated HIF-1α Content in Cells Cultured under Normoxic Conditions

**Figure 1:**
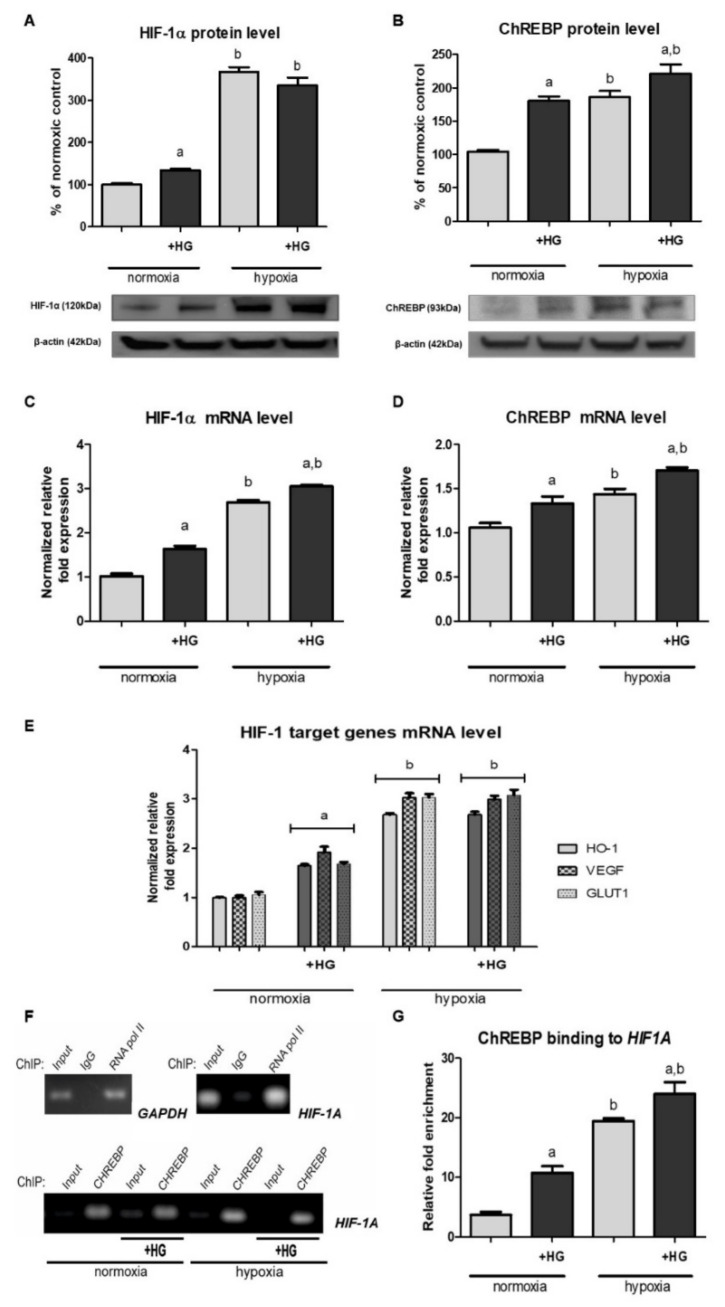
High glucose (HG) effect on HIF-1α expression ((**A**)—protein, Western blot analysis; (**C**)—mRNA, RT-qPCR analysis), ChREBP expression ((**B**)—protein, Western blot analysis; (**D**)—mRNA, RT-qPCR analysis), expression of chosen HIF-1 target genes (**E**)—mRNA, RT-qPCR analysis) and the efficiency of ChREBP binding to the promoter region of *HIF1A* gene (**F**)—end-point PCR analysis; (**G**)—relative fold enrichment of the target *HIF1A* gene over IgG sample) in HK-2 cells. Cells were cultured for 24 h under normoxic or under hypoxic (1% O_2_) conditions in the presence of normal (5.6 mM) or high (30 mM) glucose concentration. Then, 24.4 mM mannitol was added to the control media to ensure the same osmolarity. Chromatin was immunoprecipitated with anti-ChREBP, anti-RNA polymerase II (a positive control), and anti-IgG (a negative control) antibodies. PCR analyses were performed with specific primers for genes encoding GAPDH or HIF-1α, respectively, as described in 4.7. Values are means ± SEM for 3–5 experiments. Statistical significance: ^a^ *p* < 0.05 versus corresponding values in the presence of 5.6 mM glucose; ^b^ *p* < 0.05 versus corresponding values under normoxic conditions.

As shown in Figure 1A, HK-2 cells incubated in the presence of 30 mM glucose exhibited significantly higher (by ca. 40%) HIF-1α protein level than those cultured in medium with normal glucose concentration (5.6 mM). This phenomenon was observed under normoxic conditions but not in hypoxia, while high glucose-evoked increase in HIF-1α mRNA (Figure 1C) was found in case of both experimental variants. Thus, we presumed that high glucose effect on HIF-1α expression probably involves transcriptional mechanisms. However, these mechanisms might be additionally controlled at the post-transcriptional level, as under hypoxic conditions high glucose-evoked increase in HIF-1α level did not result in its elevated protein content.

### 2.2. The Expression of HIF-1 Target Genes under Normoxic Conditions Is Augmented in the Presence of High Glucose

We were intrigued if the high glucose-evoked increase in HIF-1α regulatory subunit content (cf. Section 2.1) resulted in augmented HIF-1 activity in HK-2 cells cultured under normoxic conditions. Thus, we examined the expression of three genes commonly known to be controlled by HIF-1: encoding heme oxygenase 1 (HO-1) [31], encoding VEGF [32], and encoding GLUT1 [33]. As shown in Figure 1E, under normoxic conditions, upon the addition of 30 mM glucose to experimental media, all the mRNA levels measured were increased by 60–90%, clearly suggesting that HIF-1 was activated.

### 2.3. High Glucose Increases ChREBP Expression and Its Binding to the Promoter Region of HIF1A Gene

Looking for the mechanism that might link changes in glucose availability with gene expression, we turned our attention to the transcription factor ChREBP [25,26]. As presented in Figure 1B,D, we found that high glucose led to a considerable increase in both ChREBP protein and its mRNA content in HK-2 cells, independently of oxygenation conditions.

More importantly, as shown in Figure 1F, samples immunoprecipitated with anti-ChREBP antibody exhibited a significant enrichment of the target *HIF1A* sequence, which indicated ChREBP ability to interact with the promoter region of this gene. Moreover, quantitative PCR analysis (Figure 1G) revealed that, in normoxia, this enrichment was ca. three times higher in cells cultured in the presence of high glucose in experimental medium, compared to cells cultured under normoglycemic conditions. Thus, ChIP analysis straightly confirmed the hypothesis on ChREBP binding to *HIF1A* promoter region and its crucial role in the regulation of *HIF1A* transcription in response to changing glucose availability.

### 2.4. ChREBP Knockdown Prevents the High Glucose-Evoked Increase in HIF-1α Expression

**Figure 2:**
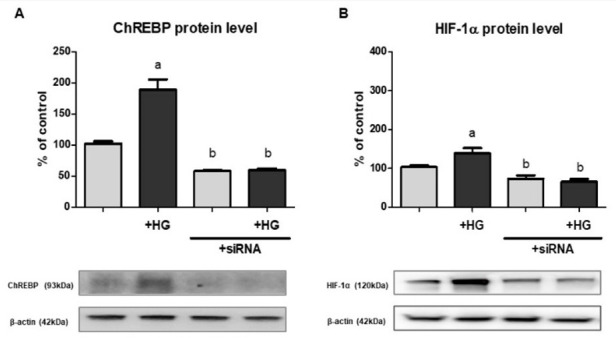
The effect of ChREBP knockdown ((**A**)—protein, Western blot analysis) on HIF-1α expression ((**B**)—protein, Western blot analysis) in HK-2 cells. Cells were cultured for 24 h under normoxic conditions in the presence of normal (5.6 mM) or high (30 mM; HG) glucose concentration. Then, 24.4 mM mannitol was added to the control media to ensure the same osmolarity. Gene silencing procedure was performed as described in detail in Section 4.5. Values are means ± SEM for 3–5 experiments. Statistical significance: ^a^ *p* < 0.05 versus corresponding values in the presence of 5.6 mM glucose; ^b^ *p* < 0.05 versus corresponding values for cells untransfected with ChREBP siRNA.

In order to ultimately confirm the postulated involvement of ChREBP in the stimulatory action of high glucose on HIF-1α expression in HK-2 cells cultured under normoxic conditions, we performed experiments applying cells having their ChREBP encoding genes silenced. As presented in Figure 2, when ChREBP protein content was experimentally decreased, the effect of high glucose on HIF-1α expression was completely abolished. Moreover, in cells with ChREBP knockdown, both in the presence of normal and high glucose concentration in experimental media, HIF-1α content was lowered by ca. 30%, compared to control cells. The above observations clearly indicated the crucial role of ChREBP in the high glucose-dependent regulation of HIF-1α expression.

### 2.5. Although High Glucose Lowers Intracellular NAD^+^/NADH Ratio, This Phenomenon Does Not Affect Either Sirtuin 1 Activity or HIF-1α Acetylation Level

**Figure 3:**
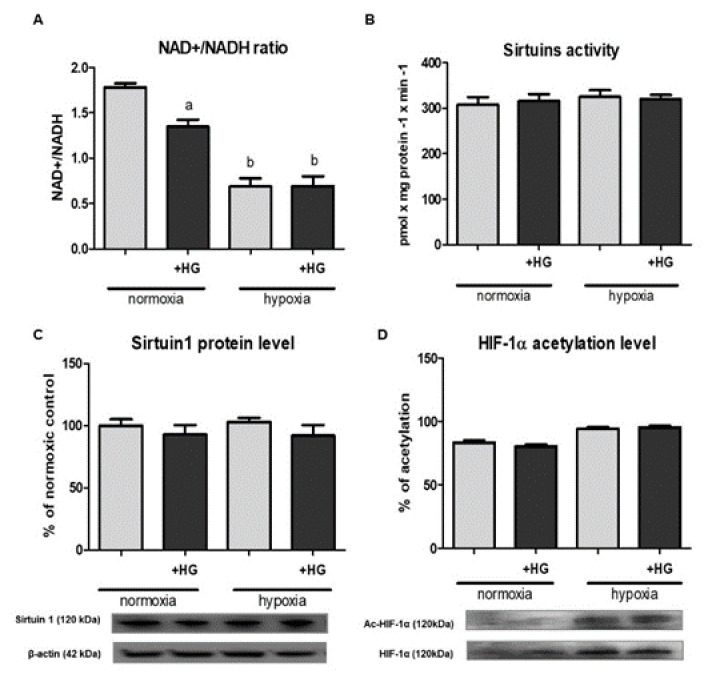
High glucose (HG) effect on intracellular NAD^+^/NADH ratio (**A**), sirtuins activity (**B**), 1 (SIRT1) expression ((**C**)—protein, Western blot analysis) and HIF-1α acetylation level ((**D**)—Western blot analysis preceded with immunoprecipitation) in HK-2 cells. Cells were cultured for 24 h under normoxic or under hypoxic (1% O_2_) conditions in the presence of normal (5.6 mM) or high (30 mM) glucose concentration. Then, 24.4 mM mannitol was added to the control media to ensure the same osmolarity. Prior to Western blot analysis, lysates intended for the determination of HIF-1α acetylation level were immunoprecipitated against HIF-1α. Following acetyl-HIF-1α analysis with anti-acetyl-lysine antibodies, the membranes were stripped and then reprobed against HIF-1α. Values are means ± SEM for 3–5 experiments. Statistical significance: ^a^ *p* < 0.05 versus corresponding values in the presence of 5.6 mM glucose; ^b^ *p* < 0.05 versus corresponding values under normoxic conditions.

The next step of our study was testing if high glucose-induced pseudohypoxia might be of importance in terms of HIF-1α stability in HK-2 cells. As shown in Figure 3A, the presence of 30 mM glucose in experimental medium caused ca. 25% decrease in the intracellular NAD^+^/NADH ratio. This effect was observed only in case of cells cultured under normoxic conditions. However, it is worth emphasizing that hypoxia itself importantly (by ca. 60%) lowered NAD^+^/NADH ratio in HK-2 cells.

To our disappointment, the changes in the intracellular NAD^+^/NADH ratio affected neither sirtuin activity (Figure 3B) nor the expression of sirtuin 1 (Figure 3C) in HK-2 cells. Consequently, HIF-1α acetylation level also remained unchanged (Figure 3D).

### 2.6. HIF-1α Content in Diabetic Proximal Tubular Cells Is Lower than That in Normal Ones but Remains High Glucose-Sensitive

**Figure 4:**
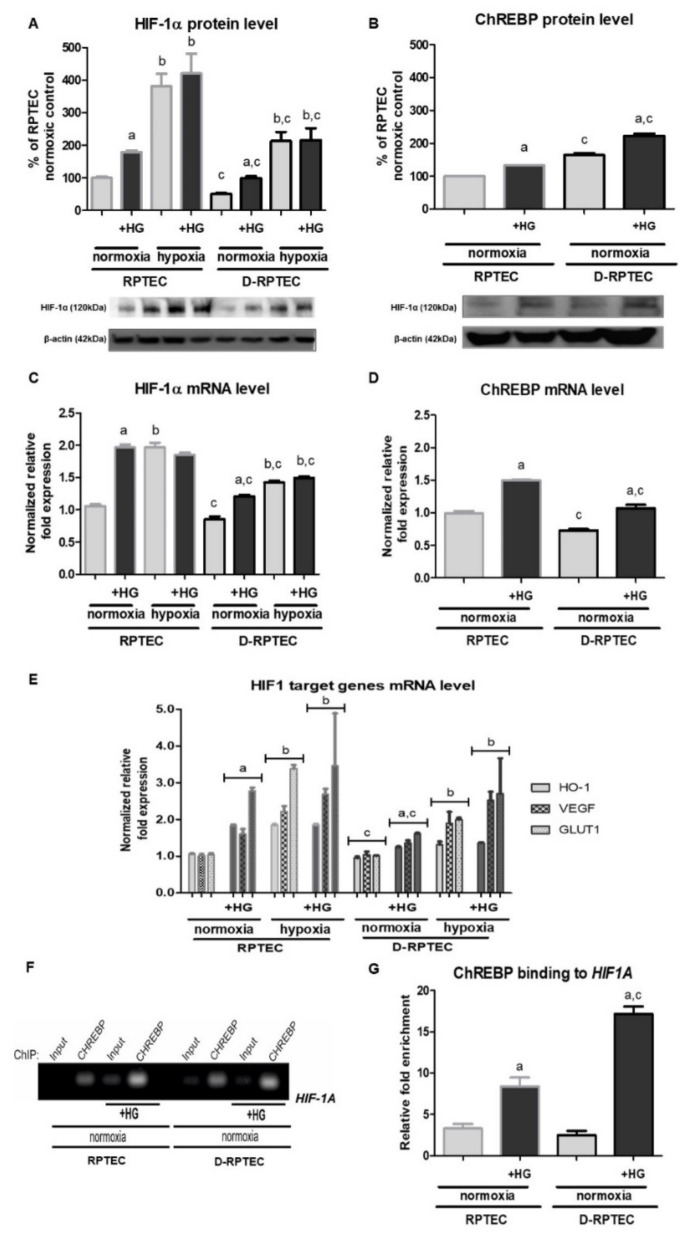
High glucose (HG) effect on HIF-1α expression ((**A**)—protein, Western blot analysis; (**C**)—mRNA, RT-qPCR analysis), ChREBP expression ((**B**)—protein, Western blot analysis; (**D**)—mRNA, RT-qPCR analysis), expression of chosen HIF-1 target genes (**E**)—mRNA, RT-qPCR analysis) and the efficiency of ChREBP binding to the promoter region of *HIF1A* gene ((**F**)—end-point PCR analysis; (**G**)—relative fold enrichment of the target *HIF1A* gene over IgG sample) in RPTEC and D-RPTEC. Cells were cultured for 24 h under normoxic or under hypoxic (1% O_2_) conditions in the presence of normal (5.6 mM) or high (30 mM) glucose concentration. Then, 24.4 mM mannitol was added to the control media to ensure the same osmolarity. Chromatin was immunoprecipitated with anti-ChREBP antibody. PCR analyses were performed with specific primes for *HIF1A* gene, as described in 4.7. Values are means ± SEM for 3–5 experiments. Statistical significance: ^a^ *p* < 0.05 versus corresponding values in the presence of 5.6 mM glucose; ^b^ *p* < 0.05 versus corresponding values under normoxic conditions; ^c^ *p* < 0.05 versus corresponding values for control cells, i.e., RPTEC.

Finally, we were interested if HIF-1α level in diabetic proximal tubular cells also changes in response to the increase in glucose concentration in experimental medium. Interestingly, diabetic cells (D-RPTEC) under all the conditions tested exhibited considerably (up to 50%) lower HIF-1α expression, both protein and mRNA (Figure 4A,C, respectively), compared to control RPTEC cells. However, under normoxic conditions, both RPTEC and D-RPTEC cells, such as HK-2 cells (cf. Section 2.1), reacted to the presence of 30 mM glucose in experimental media, considerably increasing their intracellular HIF-1α level.

As expected, media supplementation with 30 mM glucose led to augmented expression of HIF-1 target genes in either RPTEC or D-RPTEC cells cultured under normoxic conditions (Figure 4E). The effect seemed to be stronger (70–180%, depending on the gene tested) in cells withdrawn from a healthy individual, compared to that (30–70%, depending on the gene tested) observed in diabetic cells. It is also worth noticing that the changes in HIF-1 target genes expression correlated well with the changes in intracellular HIF-1α level. Under control normoxic conditions, their expression was slightly (by about 20%), but of statistical importance, lower in D-RPTEC than in RPTEC cells.

In agreement with the data obtained for HK-2 cells (cf. Section 2.3), ChREBP expression in both RPTEC and D-RPTEC cells was increased in the presence of 30 mM glucose, compared to control normoglycemic conditions (Figure 4B,D). Moreover, ChIP analysis confirmed that ChREBP binding to *HIF1A* promoter region in RPTEC and D-RPTEC cells was augmented upon 30 mM glucose addition to experimental media (Figure 4F,G).

## 3. Discussion

In this paper, we aimed to explain the mechanism responsible for the abnormally high HIF-1α content observed in epithelial cells of human renal proximal tubules cultured under normoxic conditions in the presence of 30 mM glucose.

It should be emphasized that the sparse reports on high glucose effect on HIF-1α expression in epithelial cells of renal proximal tubules are extremely divergent. In view of our data (cf. Figure 1A,C), the most challenging to discuss remain the results obtained by García-Pastor et al. [29], who found that HIF-1α response to hypoxia is impaired in HK-2 cells cultured in the presence of high glucose (25 mM) and demonstrated that hyperglycemia disrupts HIF-1α interaction with Hsp90, resulting in its accelerated degradation in proteasome. On the other hand, Zhang et al. [34] reported increased HIF-1α content (both protein and mRNA) in HK-2 cells exposed to high glucose (30 mM) under hypoxic conditions (no data for normoxia, unfortunately). Moreover, Sharma et al. [35] demonstrated stimulatory action of high glucose (30 mM) on HIF-1α protein level in HK-2 cells cultured under normoxic conditions, and a similar, although reported as statistically insignificant, effect was recently observed by Ndibalema et al. [36].

It is difficult to explain the discrepancies described above. It is probably worth taking into account that all the authors used DMEM/F12 medium supplemented with FBS (instead of Keratinocyte Serum Free Medium recommended by the cell line supplier, ATCC), but only García-Pastor et al. [29] additionally supplemented it with insulin.

In agreement with the observations made by Zhang et al. [34] for HK-2 cells incubated in 1% O_2_, we found that the exposure to high glucose could elevate either HIF-1α protein or its mRNA level (cf. Figure 1A,C). However, we observed both the effects only in normoxia; under hypoxic conditions, HIF-1α protein content remained unchanged, despite the increased mRNA level. Thus, we presumed that high glucose effect on HIF-1α expression in HK-2 involves transcriptional mechanisms, but the participation of other ones is also very probable, especially in terms of the observed dependency of high glucose effect on HIF-1α protein content on the oxygenation conditions.

Trying to find the most straightforward link between glucose availability and gene transcription, we assumed the involvement of ChREBP [25,26], demonstrating its expression in HK-2 cells (cf. Figure 1B,D). Such observations have not been made before for epithelial cells of proximal tubules, but Isoe et al. [19] found that ChREBP is able to bind to ChoRE-like sequence in the promoter region of *HIF1A* gene and suggested its crucial role in the mechanism of high glucose-augmented HIF-1α content and HIF-1 activity in mesangial cells cultured under normoxic conditions. Moreover, Park et al. [37] postulated that ChREBP-dependent increase in HIF-1α level might be responsible for the overexpression of fibrosis-related genes, e.g., encoding VEGF, fibronectin and collagen IV, in mesangial cells incubated in the presence of 25.6 mM glucose. Similarly, Chang et al. [24] reported that ChREBP binding to the promoter region of *HIF1A* gene is responsible for the rise in HIF-1α level and the activation of VEGF synthesis in retinal pigment epithelial (RPE) cells exposed to 25 mM glucose.

Interestingly, both works of Isoe et al. [19,24] emphasized that the phenomenon seems to be cell type-specific, e.g., does not occur in HeLa cells. Although Isoe et al. [19] suggested that high glucose (25 mM, 48 h) may not affect HIF-1α level in hRPTEC cells, in HK-2 cells, we observed augmented ChREBP binding to the promoter region of *HIF1A* and elevated HIF-1α mRNA level upon the exposure to high glucose (30 mM, 24 h), both under normoxic and hypoxic conditions (cf. Figure 1C,F,G). Moreover, HK-2 cells with silenced ChREBP encoding gene exhibited no high glucose effect on HIF-1α expression (cf. Figure 2), which clearly indicated the crucial role of this transcription factor in the phenomenon described in our present paper. On a different note, it is intriguing that hypoxia itself also results in the increased content and activity of ChREBP in HK-2 cells (cf. Figure 1B,D,F,G). This phenomenon is poorly explored, but it might turn out to be another important mechanism of metabolic adaptation to hypoxia.

The role of HIF-1α acetylation in the regulation of its stability [13,14,15,16,17] was the next controversial issue we studied in order to explain the differential action of high glucose on HIF-1α protein content under normoxic and hypoxic conditions (cf. Figure 1A). The hypothesis on sirtuins involvement seemed particularly attractive, as hyperglycemia results in the lowered intracellular NAD^+^/NADH ratio [8,9], while these enzymes require NAD^+^ to be fully active [10]. Additionally, there are no doubts that sirtuin 1 is able to deacetylate HIF-1α, as the direct interaction between these two proteins was confirmed by co-immunoprecipitation experiments [14,16].

Lim et al. [13] postulated the crucial role of NAD^+^/NADH ratio changes in the downregulation of HIF-1 activity by sirtuin 1. Similarly, Ryu et al. [16] suggested that augmented acetylation of HIF-1α promotes its accumulation. On the other hand, according to our latest findings [17], under hypoxic conditions it is the blunted sirtuin 1 activity that leads to the accelerated HIF-1α degradation in HK-2 cells, which was confirmed by SIRT1 knockdown. However, it could not be excluded that, under normoxic conditions, augmented acetylation of HIF-1α might have an opposite—stabilizing—effect. Although the changes in NAD^+^/NADH ratio exhibited a perfect inverse correlation with the changes in HIF-1α protein content, both of these caused by high glucose and by hypoxia (cf. Figure 1A and Figure 3A), the lowered NAD^+^/NADH ratio has no impact either on sirtuin 1 activity/content or changes in HIF-1α acetylation level (cf. Figure 3B–D).

Thus, we suggest that mechanisms other than changes in HIF-1α acetylation level are responsible for the differential effect of high glucose on this subunit content under various conditions of oxygen supply. One of the most obvious explanations might be just the superior role of oxygen availability in the control of HIF-1α level [2,11]. It seems probable that in hypoxia, under conditions of inhibited HIF-1α degradation, increased HIF-1α synthesis has a marginal effect on the final protein content.

Finally, we were especially interested in the physiological/pathophysiological importance of the high glucose-induced upregulation of HIF-1 in epithelial cells of renal proximal tubules. It is commonly accepted that hypoxia is characteristic of the kidneys of either diabetic patients, as demonstrated due to non-invasive MRI assessment [38], or animal models of the disease [6,39], and it results mainly from increased oxygen consumption, being a consequence of glomerular hyperfiltration and increased activity of sodium-dependent glucose transporters (SGLTs) and Na^+^/K^+^ ATPase [1,7]. Moreover, hypoxia is one of the factors to be blamed for the development of tubulointerstitial fibrosis, the first step leading to diabetic nephropathy [9,40,41]. However, the role of HIF-1 in this process is still an open question, i.e., it is not clear if its increased activity is responsible for the harmful effects or if it rather helps to counteract them [6,9,42]. In the present study (cf. Figure 4A,C), independently of the applied conditions of glucose and oxygen supply, we observed lower HIF-1α content in diabetic D-RPTEC cells, compared to control RPTEC cells.

Thus, we would postulate that it is rather the blunted HIF-1 response that might make diabetic proximal tubules particularly susceptible for the deleterious effects of insufficient oxygen supply. Moreover, both RPTEC and D-RPTEC cells reacted to high glucose conditions, increasing their HIF-1α content in the ChREBP-dependent manner (cf. Figure 4A,C,F,G) and upregulating HIF-1 activity, as concluded from the augmented expression of its target genes (cf. Figure 4E). The suggestion that HIF-1 might exhibit nephroprotective action under diabetic conditions seems promising in terms of the lively discussion on the therapeutic use of HIF-1 modulators. However, the issue seems extremely complicated, as, e.g., SGLT2 inhibitors, well known for their protective action in diabetic kidneys, turned out to suppress HIF-1 [43]. Moreover, according to our recent findings [5], upregulated HIF-1 activity could accelerate renal gluconeogenesis, contributing to the undesirably elevated glycemia.

Summarizing, we demonstrated that high glucose increases HIF-1α level in epithelial cells of renal proximal tubules via the mechanism including augmented ChREBP binding to the promoter region of the gene encoding this subunit. Future research is needed to establish if this phenomenon might be of importance in counteracting the consequences of the two problems diabetic kidneys suffer from, i.e., hyperglycemia and hypoxia.

## 4. Materials and Methods

### 4.1. Cell Culture

HK-2 cells (CRL-2190™, ATCC, Manassas, VA, USA; RRID:CVCL_0302), immortalized human proximal tubular cells, were cultured at 37 °C under the atmosphere of 5% CO_2_ in Keratinocyte Serum Free Medium (KSFM) supplemented with bovine pituitary extract (BPE; 0.05 mg/mL) and human recombinant epidermal growth factor (EGF; 5 ng/mL) and subcultured at 80% of confluence. The experiments were performed on the cells from the 14th passage.

Primary cells: D-RPTEC (Diseased Human Renal Proximal Tubule Epithelial Cells—Diabetes Type II) and RPTEC (Renal Proximal Tubule Epithelial Cells) originated from Lonza (Basel, Switzerland). The cells were treated precisely according to the distributor’s instructions and cultured at 37 °C under the atmosphere of 5% CO_2_ in Renal Epithelial Basal Medium (REBM™) supplemented with REGM™ SingleQuots™. The experiments were performed on the cells from the 4th passage.

High glucose conditions were achieved by supplementing the media with glucose up to 30 mM concentration. Then, 24.4 mM mannitol was added to control media containing 5.6 mM glucose to ensure the same osmolarity. Hypoxic conditions (1% O_2_) were provided due to cells incubation inside InvivO2 400 hypoxia workstation (Baker Ruskinn, Bridgend, UK).

### 4.2. Preparation of Cell Lysates

Lysates were prepared as described previously [44]. Cells cultured under hypoxic conditions were lyzed inside hypoxia workstation (cf. Section 4.1). Samples were stored at −70 °C and just before electrophoresis denatured in Laemmli buffer (5 min, 100 °C).

### 4.3. Protein Immunoprecipitation

Immunoprecipitation was performed with Protein A/G PLUS-Agarose (Santa Cruz Biotechnology, Dallas, TX, USA), according to the manufacturer’s instructions. Briefly, cell lysates (cf. Section 4.2; volumes containing 100 μg of protein) were incubated with 2 μg of anti-HIF-1α primary antibody (Santa Cruz Biotechnology, Dallas, TX, USA; cat. no sc-13515, RRID:AB_627723) for 1 h at 4 °C. Then, Protein A/G PLUS-Agarose (20 μL) was added, and the samples were incubated overnight at 4 °C. After centrifugation, the pellets containing protein-antibody-matrix complexes were carefully washed with PBS, denatured in Laemmli buffer (3 min, 100 °C), and centrifuged to remove the insoluble matrix. The supernatants dedicated for Western blot analysis were collected and stored at −70 °C.

### 4.4. Chromatin Immunoprecipitation

Chromatin immunoprecipitation (ChIP) was performed applying ChIP-IT^®^ Express Enzymatic Kit (Active Motif, Carlsbad, CA, USA) precisely according to the manufacturer’s instructions. Briefly, cells were fixed with 1% formaldehyde, and chromatin was sheared by enzymatic shearing (10 min, 37 °C). Then, immunoprecipitation procedure was performed using 5 μL of anti-ChREBP antibody (cat. no ab92809, RRID:AB_10562135) per 10 μg of chromatin. ChIP-IT^®^ Control Kit—Human (Active Motif, Carlsbad, CA, USA) was used as a quality control. Both mRNA purification and PCR analysis were performed as described in Section 4.7.

### 4.5. Gene Silencing

For the transient silencing of the gene encoding ChREBP, HK-2 cells were transfected with TransIT-TKO^®^ Transfection Reagent (Mirus Bio LLC, Madison, WI, USA,), according to the manufacturer’s instructions. ChREBP siRNA (h) (Santa Cruz Biotechnology, cat. no. sc-3861) was used as target siRNA and applied at 100 nM concentration, as recommended by the manufacturer. SignalSilence^®^ Control siRNA (Unconjugated) (Cell Signaling Technology, Danvers, MA, USA; cat. no. #6568) served as a negative control. The efficiency of the silencing was confirmed by Western blot analysis (cf. Section 4.6) of ChREBP protein content in cells lyzed 48 h after transfection. There was no difference in ChREBP expression in intact cells and in cells transfected with negative control siRNA.

### 4.6. Western Blot Analysis

Samples (15 μg protein/lane) were applied to polyacrylamide gels (10%; Lonza, Basel, Switzerland), or made according to Reference [45], electrophoresed, electroblotted to polyvinylidene fluoride (PVDF) membranes (BioRad, Hercules, CA, USA), and incubated with proper antibodies, as described previously [17]. β-actin was used as a loading control [17].

Protein detection was performed by the enhanced chemiluminescence (ECL) method using ChemiDoc™ Imaging System (BioRad, Hercules, CA, USA). The density of the bands was analyzed by Image Lab software (BioRad, Hercules, CA, USA; RRID:SCR_014210). Electrophoresis and electroblotting were performed using, respectively, MiniProtean Tetra System and TransBlot System (BioRad, Hercules, CA, USA).

### 4.7. PCR Analysiss

Total RNA was extracted with Universal RNA Purification Kit (EURx, Gdansk, Poland), according to manufacturer’s instructions for cell cultures (with DNase digestion), and eluted with 70 μL of water. Directly following the isolation, RNA quality was checked with NanoDrop spectrophotometer (Thermo Scientific Inc., Waltham, MA, USA). RNA was stored at −80 °C and thawed only shortly prior to the experiment. The primers for mRNAs detection were designed, respectively, according to: HIF-1α, β-actin, VEGF—[32]; RPL13A—[46]; HO-1—[31]; and GLUT1—[33].

Primers hybridizing with *HIF1A* promoter region were designed according to Isoe et al. [19]. The pair for GAPDH was supplied with ChIP-IT^®^ Control Kit—Human (Active Motif).

Expression analysis was conducted by quantitative PCR with MyGo Pro Real-Time PCR thermocycler (IT-IS International Ltd., Middlesbrough, UK), using SensiFAST™ SYBR^®^ Green MasterMix (Bioline, London, UK) and recommended thermal profile (45 cycles). Following amplification, a melt curve was performed in the 60–95 °C range, with 0.5 °C steps.

Relative gene expression in each sample was calculated with My Go Pro analysis software v.3.3 (IT-IS International Ltd., Middlesbrough, UK), normalized to reference genes (β-actin and RPL13A) and scaled to the calibrator sample (obtained in the absence of melatonin). Intra-assay variation was evaluated by calculating SEM errors of sample replicates.

### 4.8. NAD^+^/NADH Ratio Determination

Intracellular NAD^+^/NADH ratio was estimated applying NAD/NADH Quantitation Colorimetric Kit (BioVision Inc., Milpitas, CA, USA), according to the manufacturer’s instructions. Prior to the final determinations homogenates (ca. 2 × 10^5^ cells per sample) were deproteinized by filtrating though 10 kDa cut off spin filter (10 kD Spin Column, BioVision Inc., Milpitas, CA, USA). Absorbance (450 nm) was measured using Victor3 plate reader (PerkinElmer, Waltham, MA, USA).

### 4.9. Sirtuin Activity Determination

Sirtuin activity was measured with Sirtuin Activity Assay Kit (BioVision Inc., Milpitas, CA, USA), basing on fluorimetric determination of fluorochrome (AFC) realized from acetyl-p53-AFC in reaction catalyzed by sirtuins.

Both cells (ca. 2 × 10^6^ per sample) homogenization and all the following steps were performed precisely according to the manufacturer’s instructions. Fluorescence (Ex/Em = 400/505 nm) was measured using Infinite M200 PRO plate reader (Tecan Group Ltd., Männedorf, Switzerland).

### 4.10. Protein Determination

All the determinations of protein content were performed spectrophotometrically according to Bradford [47].

### 4.11. Antibodies and Chemicals

The antibodies originated, as follows, from: Cell Signaling Technology (Danvers, MA, USA)—anti-HIF-1α (cat. no. #36169, RRID:AB_279909), used for Western blot), anti-ChREBP (cat. no. #58069, RRID:AB_2799539, used for Western blot), anti-SIRT1 (cat. no. #2310, RRID:AB_561272), anti-acetylated-lysine (cat. no. #9441, RRID:AB_331805) and anti-rabbit IgG (cat. no. #7074, RRID:AB_2099233); Santa Cruz Biotechnology (Dallas, TX, USA)—anti-HIF-1α (cat. no. sc-13515, RRID:AB_627723, used for immunoprecipitation); Abcam (Cambridge, UK)—anti-ChREBP (cat. no ab92809, RRID:AB_10562135, used for ChIP) and anti-beta-actin conjugated with HRP (cat. no. ab49900, RRID:AB_867494).

The oligonucleotides: ChREBP siRNA (h) (cat. no. sc-38617) and SignalSilence^®^ Control siRNA (Unconjugated) (cat. no. #6568) were from, respectively, Santa Cruz Biotechnology (Dallas, TX, USA) and Cell Signaling Technology (Danvers, MA, USA).

Sirtuin Activity Assay Kit (Fluorometric) and NAD/NADH Quantitation Colorimetric Kit originated from BioVision Inc. (Milpitas, CA, USA). ChIP-IT^®^ Express Enzymatic Kit and ChIP-IT^®^ Control Kit—Human were manufactured by Active Motif (Carlsbad, CA, USA). RNA purification kit and PCR chemicals originated from, respectively, EURx (Gdansk, Poland) and Bioline (London, UK). ECL reagent—Westar Supernova was from Cyanagen (Bologna, Italy). A/G PLUS-Agarose was from Santa Cruz Biotechnology (Dallas, TX, USA). TransIT-TKO^®^ Transfection Reagent originated from Mirus Bio LLC (Madison, WI, USA). Keratinocyte-SFM (1X) kit was from Gibco (Carlsbad, CA, USA). REGM™BulletKit™ (CC-3190; containing REBM™ and REGM™ SingleQuots™) originated from Lonza (Basel, Switzerland). All other chemicals were purchased from Sigma Chemicals (St. Louis, MO, USA).

### 4.12. Expression of Results

The significance of the differences was estimated using One-way ANOVA with Bonferroni post-test. Values are expressed as means ± SEM for 3–5 separate experiments.

## Data Availability

The data are available at request from the authors.
